# Physiological strength electric fields modulate human T cell activation and polarisation

**DOI:** 10.1038/s41598-019-53898-5

**Published:** 2019-11-26

**Authors:** Christina E. Arnold, Ann M. Rajnicek, Joseph I. Hoare, Swechha Mainali Pokharel, Colin D. Mccaig, Robert N. Barker, Heather M. Wilson

**Affiliations:** School of Medicine, Medical Sciences & Nutrition, University of Aberdeen, Foresterhill, Aberdeen, AB25 2ZD UK

**Keywords:** T cells, Medical research

## Abstract

The factors and signals driving T cell activation and polarisation during immune responses have been studied mainly at the level of cells and chemical mediators. Here we describe a physical driver of these processes in the form of physiological-strength electric fields (EFs). EFs are generated at sites where epithelium is disrupted (e.g. wounded skin/bronchial epithelia) and where T cells frequently are present. Using live-cell imaging, we show human primary T cells migrate directionally to the cathode in low strength (50/150 mV/mm) EFs. Strikingly, we show for the first time that EFs significantly downregulate T cell activation following stimulation with antigen-activated APCs or anti-CD3/CD28 antibodies, as demonstrated by decreased IL-2 secretion and proliferation. These EF-induced functional changes were accompanied by a significant dampening of CD4^+^ T cell polarisation. Expression of critical markers of the Th17 lineage, RORγt and IL-17, and the Th17 polarisation mediator phospho-STAT3 were reduced significantly, while STAT1, ERK and c-Jun phosphorylation were comparatively unaffected suggesting STAT3 modulation by EFs as one mechanism driving effects. Overall, we identify electrical signals as important contributors to the co-ordination and regulation of human T cell functions, paving the way for a new research area into effects of naturally occurring and clinically-applied EFs in conditions where control of T cell activity is paramount.

## Introduction

During the adaptive immune response, activated CD4^+^ T cells expand and differentiate into different functional subtypes in the peripheral tissues that play an essential part in immune protection^1,2^. For example, Th1 cells help macrophages destroy intracellular pathogens and viruses and can drive chronic inflammation, Th2 cells recruit eosinophils to attack parasites and extracellular pathogens and are involved in allergic conditions and Th17 cells recruit neutrophils to clear fungi and other microbial pathogens at mucosal surfaces, but mediate autoimmune disorders. Regulatory T cells (Treg) also play a pivotal role in immune homeostasis by suppressing excessive immune responses and maintaining peripheral tolerance^2^. Differentiation of naïve CD4^+^ T cells into effector subtypes with distinct cytokine profiles and physiological roles is a tightly regulated process, the imbalance of which can lead to immune-mediated disease. Defining the mechanisms and factors underlying CD4^+^ T cell activation and differentiation is therefore of strong clinical relevance. CD4^+^ T cells are activated in secondary lymphoid tissues such as lymph nodes but can also be stimulated subsequently by antigen presenting cells after migrating to and entering sites of inflammation. T cells are therefore exposed to and integrate signals from a plethora of microenvironmental stimuli throughout the period of activation.

T cell activation, proliferation and differentiation has been extensively studied in the context of contact-dependent mechanisms and cytokines and other chemical mediators binding to receptors. However, physical factors are now being recognized for their role in driving T cell functions. Lymphocytes can respond to applied physiologically relevant direct current electric fields (EFs) by migrating toward the cathode of the fields (electrotaxis) in both *in vitro* and *in vivo* settings^3,4^. Endogenous direct current EFs have been demonstrated in development, regeneration and pathology^5–7^. Endogenous EFs arise in lesioned epithelia because their barrier function is compromised. One consequence of an epithelial barrier is the establishment of a natural trans epithelial voltage difference that arises from the polarised distribution and functional variation of ions, ion pumps and ion channels on either side of the epithelial cells. Injuries that breach the seal across epithelial layers e.g. wounding or physical disruption of the bronchial epithelium generate a localized endogenous EF that plays a pivotal role in the healing process^8–13^. EFs have been measured directly at sites where the epithelium is disrupted, and T cells are present e.g. bronchial epithelium in asthma and skin epithelia in wounds^9,14^. As well as driving directional cell migration, EFs have been shown to influence cellular functions, such as increased phagocytosis in macrophages and neurite growth during development^15,16^. However, the role of EFs in T lymphocyte function is less well documented. The purpose of the work was to determine how physiological strength EFs influence CD4^+^ T cell activation (IL-2 secretion and proliferation) and polarisation (Th signature cytokine secretion and transcription factor activation) in shaping immune responses, and to identify the mechanisms that exert any such effects. Our results identify the responses and novel pathways that are activated in CD4^+^ T cells by physiological strength EFs and could have important clinical implications for T cell mediated diseases.

## Results

### EF exposure suppresses activation and proliferation of stimulated T cells

**Figure 1:**
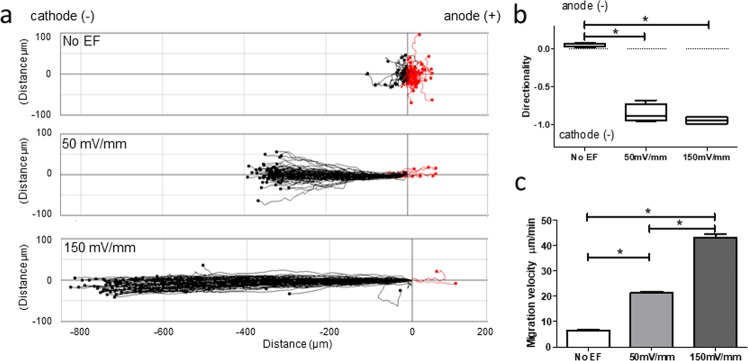
Human T lymphocytes migrate directionally to the cathode. Migrational displacement (Td) of human T lymphocytes migrating with no EF exposure or EF of 50 and 150 mV/mm over a 15 minute period, with 4 visual fields per condition (**a**). Each point represents the final position of a single cell relative to their starting position at 0 h at the origin. Cell migration was recorded by tracking Td between frames at 30 second intervals. Directedness of T cell migration (**b**). Velocity of cell migration (**c**). Data are representative of independent experiments from 3 individual lymphocyte preparations, 100 cells analysed per donor per condition. Paired t test; *P < 0.001.

Human lymphocytes have been shown previously to migrate to the cathode upon exposure to EFs^3,4^. Using our EF-cell migration experimental protocol and EF strengths of 50 and 150 mV/mm, similar to those found in wounded skin^9,14^ or airway epithelia^8^, we confirmed the responsiveness of T lymphocytes to an applied EF and consequently their striking preferential cathodal migration (Fig. 1a and Supplementary Video 1). Most lymphocytes (>80%) migrated to the cathode at both EF strengths; by contrast, migration of non-EF-exposed control cells did not show a significant directional preference and migrated randomly. The directedness of migration (negative value indicates cathodal migration) was skewed heavily by EF exposures of 50 and 150 mV/mm compared to cells without an EF (50 mV/mm, −0.85 ± 0.06; 150 mV/mm, −0.94 ± 0.02; no EF, 0.05 ± 0.01; P < 0.0001; Fig. 1b). Directed migration was so marked, it gave the appearance of virtually all cells moving along a straight line represented by the EF vector. This was not merely due to electrophoresis of whole cells due to the EF, as most cells are negatively charged and thus would move electrophoretically to the anode. The velocity of T cell migration also was enhanced greatly by EF stimulation, increasing by 3-fold and by a remarkable 6-fold at 50 and 150 mV/mm respectively (50 mV/mm, 21.44 ± 0.42 µm/min; 150 mV/mm, 43.16 ± 1.18 µm/min; no EF control 6.58 ± 0.23 µm/min; P < 0.0001; Fig. 1c). The cathodal-directed T cell migration was voltage dependent as supported by the higher percentage/faster rate of migration in a field strength of 150 mV/mm as compared to 50 mV/mm.

**Figure 2:**
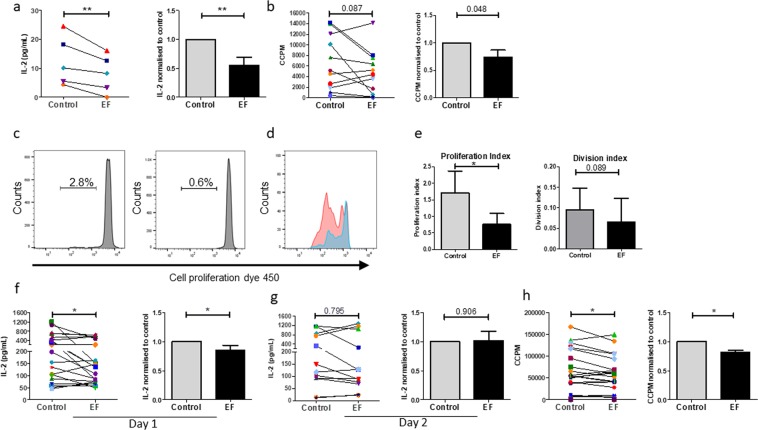
EF exposure induces changes in PBMC and T cell activation and proliferation. Human PBMCs lymphocytes were activated by PPD recall antigen (5 µg/ml) and LPS (1 µg/ml), with (EF) or without (Control) exposure to EF 150 mV/mm for 4 h. (**a**) Levels of IL-2 in culture supernatants were determined by ELISA 36 hours following EF-exposure. (**b**) Cell proliferation was determined on day 5 post EF-exposure by ^3^H-thymidine incorporation. Human T lymphocytes were stimulated with anti-CD3/CD28 antibodies. (**c**) Example of proliferation histograms for PPD/LPS stimulated PBMCs without and with EF as assessed by cell proliferation dye, CellTrace Violet. The percentage of reactive cells proliferating are shown. (**d**) A direct comparison of the mean number of cell divisions by PMBCs activated by PPD/LPS with and without EF exposure. (**e**) The change in proliferation index (total number of divisions divided by the number of cells that went into division) and division index (mean number of cell divisions that a cell in the original population has undergone) for PPD/LPS activated cells with and without EF exposure (n = 3). Levels of IL-2 in culture supernatants were determined by ELISA (**f**) 18 hours (day 1) and (**g**) 36 hours (day2) following EF-exposure. (**h**) T cell proliferation was determined on day 3 post EF-exposure by ^3^H-thymidine incorporation. For a, b, f, g and h, data plotted represent individual donor values and right panels represent the normalised data determining the ratio of EF-exposed cells to control cells for each donor; mean values ± SEM. *p < 0.05, **p < 0.01.

We extended our finding that T cells were responsive to EFs by defining whether a physiological strength EF could influence the activation of human primary T cell-induced immune responses, as determined by levels of IL-2 secretion and by the extent of proliferation. To induce a physiological antigen-specific immune response, PBMCs were activated with the recall antigen, purified protein derivative (PPD) and LPS^17^, immediately prior to EF-exposure for 4 hours and compared to cells with no EF (control). Secretion levels of IL-2 in culture supernatants, measured 36 h after EF-exposure, showed a significant reduction in EF-exposed cultures as compared to control cultures (Fig. 2a). Analyses of proliferative responses, as assessed by DNA synthesis 5 days post EF, also showed a decrease in EF-exposed cultures compared to control unexposed cells in the majority of donors (Fig. 2b, 7 donors showed a decrease and 4 an increase). To analyse this decrease further, the percentage of cells undergoing proliferation and the number of cell divisions was determined. As expected, only a small proportion of cells responded to PPD^17^ and this was decreased following EF exposure (mean values; control, 2.33 ± 0.49%, with EF, 1.16 ± 0.49%; p < 0.05; Fig. 2c–e). The division index, demonstrating the mean number of cell divisions that a cell in the original population has undergone, was also reduced following EF exposure (control, 0.095 ± 0.05%, with EF 0.07 ± 0.06; p = 0.089) as was the proliferation index as a measure of divisions by responder cells only (1.71 ± 0.32; EF 0.75 ± 0.32; p < 0.05). These results suggest that EFs affect changes in early signaling events.

The effects of EFs on proliferation could be influenced indirectly via other immune cells within the PBMC culture rather than on T cells only. To test whether EFs affect T cell responses directly, they were activated with anti-CD3/CD28 antibodies, followed by immediate EF-exposure for 4 hours (EF), or were left without EF (control). This avoided any potential effects of APCs that could influence results observed. Secretion of IL-2 was measured on day 1 and day 2 following EF-exposure, while proliferation was measured on day 3. The results demonstrated an overall significantly reduced level of IL-2 in the supernatant of activated T cells, following 18 hours of culture post EF exposure (Fig. 2f), with the majority (›75%) of individual donor T cells demonstrating a decrease in IL-2 production. However, this significant difference was lost at day 2 post EF exposure (Fig. 2g). This activation at an early time point suggests that the EF effects are short term or that to be sustained they require continuous EF exposure. In line with the early decrease in IL-2, analysis of T cell proliferation also showed a modest but significant decrease in EF-exposed cultures compared to control unexposed cells (Fig. 2h). These data indicate that EFs modulate CD4^+^ T cell activation directly, without influences from other cell types in the PBMC cultures.

**Figure 3:**
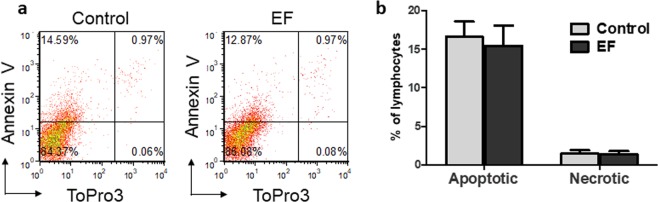
EF exposure does not induce cell death. On day 3 following EF-exposure T lymphocytes were harvested and stained with ToPro-3 (viability stain) and annexin V (apoptosis stain). Cells exposed to an EF did not show a significant change in either marker, indicating the absence of lethal effects of EFs. (**a**) Flow cytometry plots of one representative donor. (**b**) Analysis of the percentages of apoptotic and dead T cells, mean values ± SEM for n = 3.

An apparent decrease in proliferation (Fig. 2h) can also represent a lower total cell number as a result of increased cell death, so the influence of physiological strength EFs (150 mV/mm) on T cell viability was determined. T cells were harvested on day 3 following EF-exposure and stained with ToPro3 (necrotic cell stain) and Annexin V (apoptotic cell stain). No significant change in the percentages of apoptotic or necrotic cells was observed between control and EF exposed cells (Fig. 3a,b), suggesting that changes detected in T cells result from specific EF-induced signaling events and their downstream effects on proliferation.

### EFs exposure decreases T cell cytokine secretion

**Figure 4:**
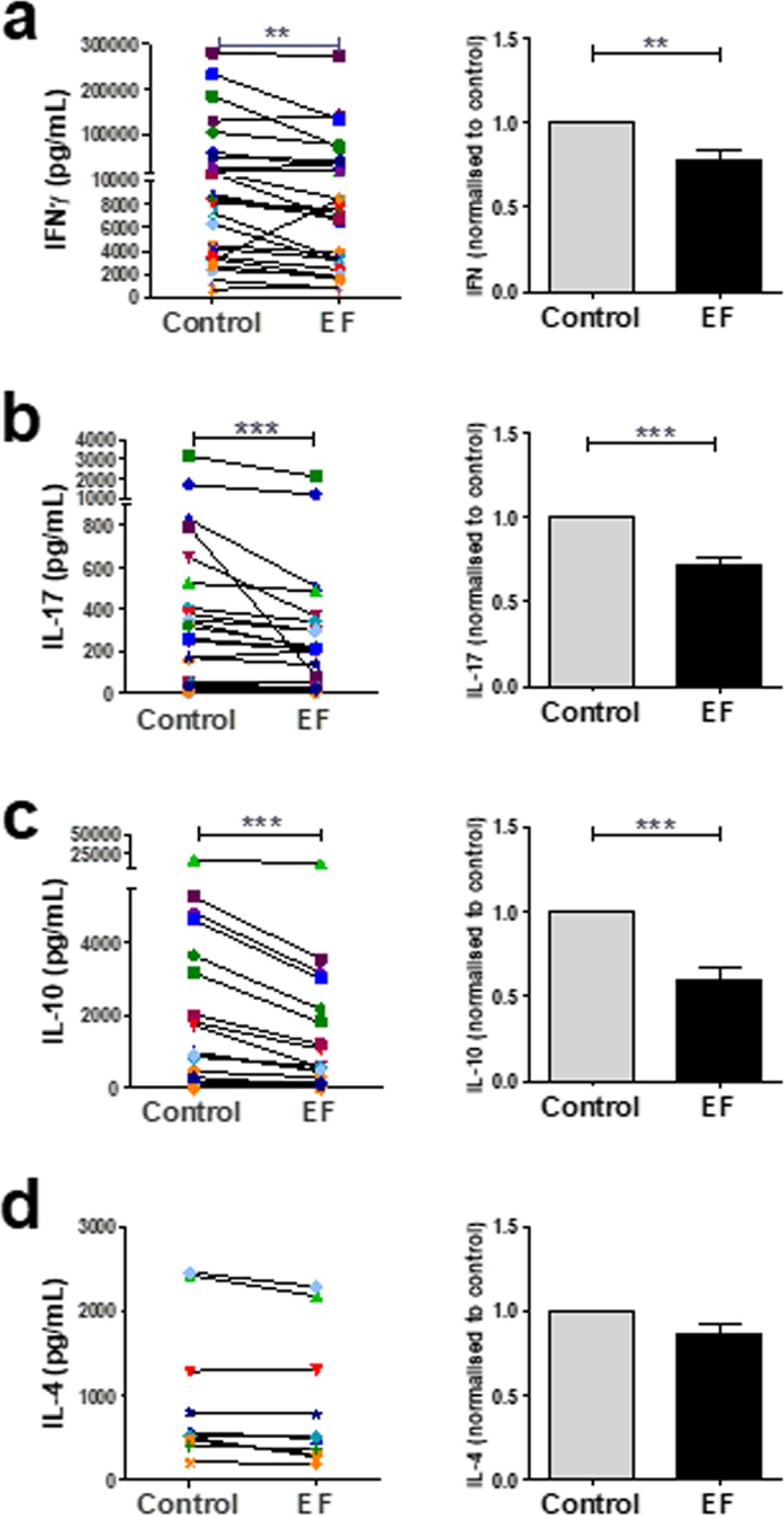
EFs attenuate T cell-derived cytokine secretion. Human T cells (2.5 × 10^6^) were stimulated with anti CD3/CD28 antibodies and EF applied for 4 hours at 150 mV/mm. Supernatants were analysed for cytokine production 3 days post EF application. Data plotted represent individual donor values (left panel) and right panels represent the normalised data determining the ratio of EF-exposed cells to control cells for each donor; mean values ± SEM. **p < 0.01; ***p < 0.005.

To further characterise the effect of EF exposure on T cell functions, cells were activated with anti-CD3/CD28 antibodies and cultured in the presence or absence of EFs (4 hours at 150 mV/mm) to determine changes in the production of the major T cell subset cytokines IFNγ (Th1), IL-4 (Th2), IL-17 (Th17), and IL-10 (T reg cells) 3 days post activation. EF exposure reduced IFNγ secretion levels in the majority of donors tested (23 of the 27 donors; Fig. 4a, p < 0.01) with an overall significant decrease in levels detected. The levels of IL-17 were attenuated markedly in EF-exposed cultures for every donor except one (n = 27), resulting in an overall significant reduction (Fig. 4b; p < 0.005). The levels of IL-10 were likewise reduced in 25 of the 27 of donors in EF-exposed cultures (Fig. 4c; p < 0.005), however, levels of IL-4 were not significantly affected by EF stimulation (Fig. 4d). Similar results were observed when PBMCs were activated with PPD recall antigen and LPS to induce Th cell polarising cytokines in the presence or absence of EFs, with levels of IL-17 most significantly changed by EF exposure. (Supplementary Figure 1). These data show that in addition to attenuating T cell proliferation, EFs modulate CD4^+^ T cell polarisation.

### EFs affect expression of lineage specific transcription factors in T cells

**Figure 5:**
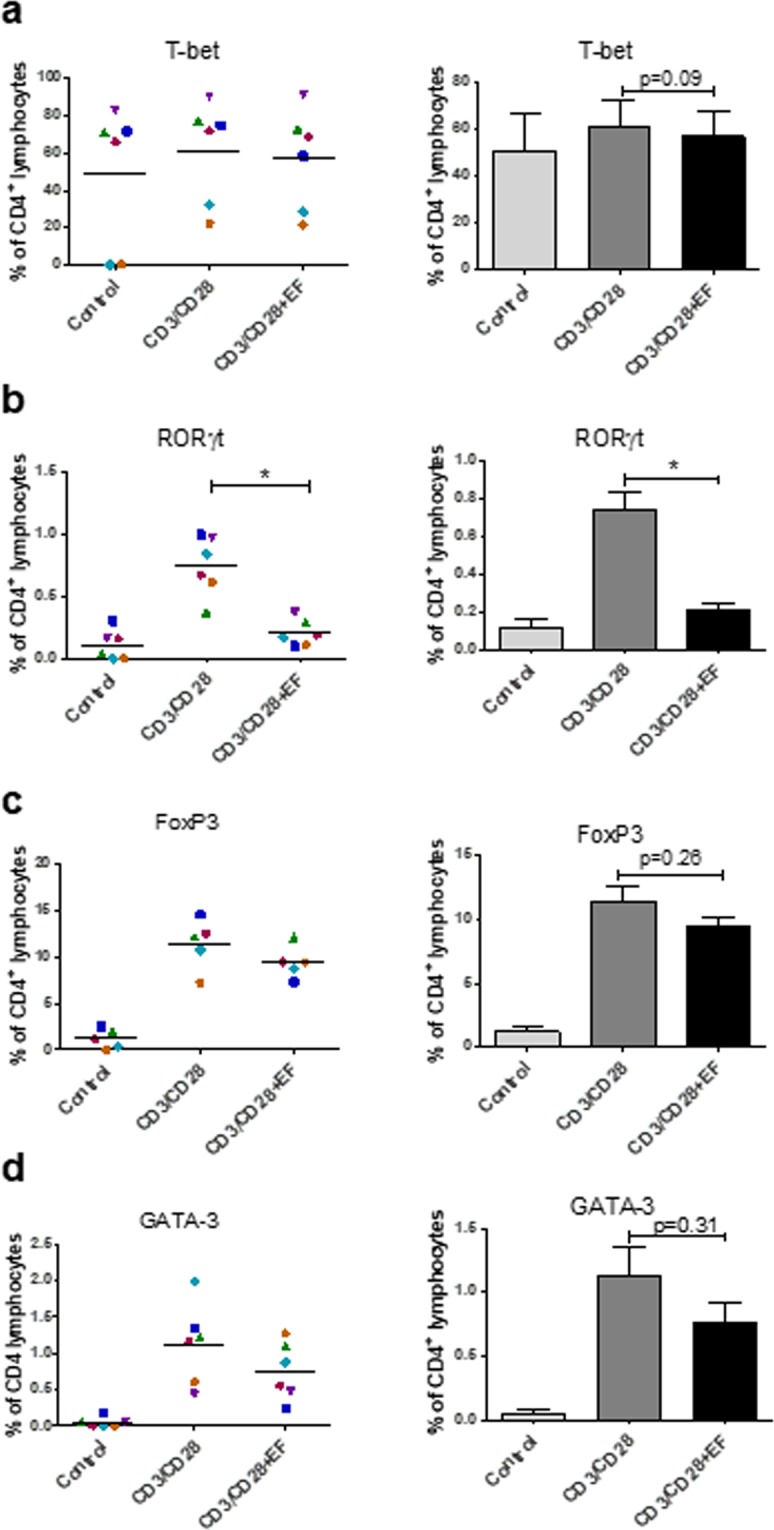
Effect of EF on expression of lineage specific transcription factors. T cells were left unstimulated (control) or activated with anti CD3/CD28 antibodies (CD3/CD28). Following exposure to EFs (CD3/CD28 + EF), T cells were harvested and 3 days post EF stained for the lineage specific transcription factors (**a**) T-bet (Th1), (**b**) RORγt (Th17), (**c**) FoxP3 (Treg) and (**d**) GATA-3 (Th2). Data plotted represent individual donor values and right panels show the mean values ± SEM; n = 6 separate T cell cultures; *p < 0.05.

T cell lineage specific transcription factors are responsible for the induction of specific genes, including those leading to the expression of the cytokines^18^ that characterise different subsets. To confirm that changes in cytokine production in response to EFs as measured above reflect effects on distinct T cell polarisation subsets, the cell populations expressing lineage-specific transcription factors were analysed. Expression of T-bet (Th1), RORγt (Th17), GATA-3 (Th2) and FoxP3 (T reg cells) was determined by intracellular staining and by flow cytometric analysis, 3 days post activation and EF exposure. The percentage of T-bet expressing cells was only marginally increased by anti-CD3/CD28 antibody activation (Fig. 5a). EF exposure resulted in a non-significant decrease in the number of activated T-bet expressing cells. By contrast, the percentage of T cells expressing RORγt, a Th17 associated transcription factor, increased significantly upon activation with anti CD3/CD28 antibodies (Fig. 5b). The percentage of RORγt T cells was, however, decreased strikingly (p < 0.05) in EF-exposed cultures compared to non-EF exposed, activated cells, with expression levels similar to non-activated T cells (Fig. 5b). The decrease in RORγt expressing T cells in EF-exposed cultures reflected the significant decrease in IL-17 levels after the same treatment (Fig. 4f). The percentage of T cells expressing FoxP3, which is indicative of Treg cells, was not decreased significantly following EF exposure, despite reduced IL-10 levels (Fig. 5c). Moreover, the level of GATA-3^+^ cells, indicative of Th2 polarisation, was overall not reduced significantly, although the majority of donors showed a lower percentage of GATA-3^+^ cells in EF-exposed cultures (Fig. 5d).

Therefore, despite the statistically significant decreases in Th1, Th17 and Treg specific cytokines, only the percentage of Th17 phenotypic cells (RORγt) was reduced significantly by EFs. This suggested that Th17 polarisation is the differentiation pathway most sensitive to the effects of EFs.

### EF exposure decreases STAT3 phosphorylation

**Figure 6:**
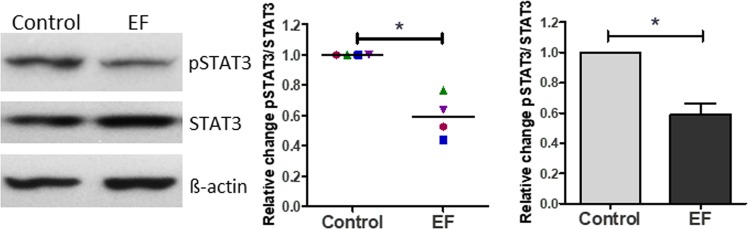
EF exposure results in a decrease in STAT3 phosphorylation in T cells. T cell derived protein was isolated 18 hours following 4 h EF-exposure (150 mV/mm) and analysed for STAT3, pSTAT3 and β-actin expression levels by Western Blotting. p-STAT3:STAT3 band intensity, was determined by densitometry and expressed as the ratio of p-STAT3 to STAT3. Shown as mean ± SEM of independent experiments from 4 individual human T cell preparations; *p < 0.05. Blots shown are from the same gel. The blots were stripped after probing for p-STAT3 and reprobed with β-actin. The full-length blots are presented in Supplementary Figure 3.

To understand the mechanism by which EFs could alter T cell responses, we next tested the activation of potential intracellular pathways PI3K (AKT), ERK, cJun, STAT1 and STAT3 that could direct the T cell responses. The pathways were analysed following EF exposure and 18 h T cell activation by anti CD3/CD28 antibodies rather than immediately after EF exposure, to define the effects on downstream signalling that influences T cell subset polarisation as opposed to EF induced effects *per se*. STAT3 activity is a major driver of Th17 polarisation, while STAT1 activation upregulates Th1^2^. The level of p-STAT3 was reduced significantly in T cells 18 h after they were exposed to an EF (Fig. 6), in line with its role in driving RORγt expression and expression of IL-17 and the IL-23 receptor, which are all essential for Th17 polarisation^2,18^. However, this decrease was not observed 3d after EF exposure (data not included) demonstrating the effect was not sustained long term. By contrast, no change in STAT1 activation was noted (Supplementary Figure 2), paralleling the lack of significant changes in T-bet expression. No consistent or significant change in activation of the other pathways tested was noted after EF exposure (Supplementary Figure 2) demonstrating only defined pathways are altered at the time point examined. Taken together, the results show that an externally applied EF reduces Th17 polarisation significantly and selectively, an effect that is likely to be mediated, at least in part, by EF-induced down-regulation of STAT3 phosphorylation.

**Figure 7:**
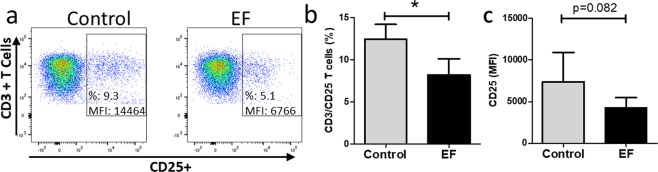
EFs can suppress the percentage of CD3+, CD25+ T cells. The proportion of CD3^+^ CD25^+^ T cells was detected by using flow cytometry. (**a**) Representative example of dot plots showing CD25^+^ T cells as a proportion of viable CD3^+^ cells and the CD25 mean fluorescent intensity (MFI) 5 days after activation with PPD and LPS, without and with EF. (**b**) Mean percentage ± SEM of CD3^+^ CD25^+^ T cells, 5 days after activation, with and without EF exposure. (**c**) The MFI of CD25 in CD3 + T cells, n = 3 separate T cell cultures; *p < 0.05.

STAT3 activation is required for efficient T cell proliferation through IL-2 receptor α (CD25) induction and STAT3 deficient T cells are partially defective in IL-2 induced proliferation^19^. To define if the decrease in STAT3 activity could relate to a potential mechanism by which proliferation is reduced, the next experiments investigated whether EF exposure impairs the antigen-induced upregulation of CD25 on T cells. PBMCs activated by PPD and LPS were exposed to EF for 4 h and expression of CD25 determined and compared to unexposed cells 5 days later. EF exposure resulted in a significant decrease in the proportion of CD25 expressing CD3 + cells and those with upregulated CD25, expressed overall lower surface levels (Fig. 7a–c) relating to the decrease in STAT3 activity and proliferation with EF.

## Discussion

We show for the first time that direct current EFs, of similar strength to those generated naturally *in vivo*, e.g. in wounded skin^9,14^ or airway epithelia^8^, are key signals that regulate early T cell activation and polarisation *in vitro*. Most previous studies have focused on the cell contact dependent mechanisms, for example, antigen presentation signals and chemical signals, including cytokines, in driving these processes, with much less known about how physical factors, such as extracellular EFs, influence T cell function. Here we show that physiological strength EFs not only regulate migration of T cells but also their proliferation, cytokine output and STAT3 signaling pathways that drive functional responses. Therefore, EFs are important, but generally unresearched physical cues regulating the strength of T cell responses, and are highly relevant to inflammatory, atopic and autoimmune disorders, where T cell activity is exaggerated.

Endogenous EFs are generated naturally at sites of injury that disrupt epithelial cell barriers and this is key in driving healing processes^8–10,13^. This effect is partially attributed to the strong directional guidance cues for cellular migration, for example, of fibroblasts and keratinocytes drawn in to close the wound^13,20^. Here, we show externally applied EFs of a physiological range also induce directional migration of T cells towards the cathode (wound site). The directional migration could be considered to be driven partially by flow effects (electroosmosis) although this would be minimal^21^. Of crucial importance, we see non-linear cell tracks and also, some cells reverse direction in the EF with non-linear tracks, therefore we do not believe that unidirectional electro-osmosis could be the reason they move directionally. This EF-induced migration confirms previous reports demonstrating cathodal migration of lymphocytes in response to EF exposure *in vitro* and *in vivo*^3,4^. Thus, EFs could potentially help position T lymphocytes in areas where epithelium is disrupted to assist the healing process.

IL-2 secretion is a critical and early landmark in the activation program of CD4^+^ T cells. The downregulation of IL-2 secretion following EF exposure suggests that early signals are influenced and consequently result in the attenuated proliferation observed in our studies. We show this downregulation effect of IL-2 secretion and proliferation is a direct result of the EF and not due to EF-induced lymphocyte apoptosis or toxic effects. The inhibitory effect of EF exposure on IL-2 production is much greater when APCs are used to activate the T cells than when the T cells are activated via anti-CD3/CD28 antibodies. This could be because anti-CD3/CD28 delivers a much stronger activating signal by engaging all T cell receptors, as opposed to the small number of cognate peptide/MHC on the surface of an APC. An alternative explanation is that EFs also have an inhibitory effect on the ability of APC to process and present antigens, upregulate co-stimulatory molecules, or produce cytokines^15^.

For the body’s adaptive immune system to elicit an effective immune response and clear pathogens effectively, CD4^+^ helper T cells differentiate into particular subtypes^1,2^. We demonstrate a clear dampening of the Th1, Th17 and Treg associated cytokines, IFNγ, IL-17 and IL-10, but not Th2 associated IL-4 from activated T lymphocytes, suggesting a broad attenuation of immune responses by EF exposure. It is of interest that magnetic fields also induced a decrease in cytokine secretion (IFNγ) by mitogenic (phytohaemagglutinin) activated T cells, further demonstrating the importance of physical stimuli in influencing T cell activity^22^. When analysing differentiation based on Th cell signature transcription factors in our study, RORγt (Th17 polarisation) was most affected. Thus, while the trend is for a range of response types to be suppressed by EF, the inhibition is more marked for some, most notably the Th17 subset.

The ability of EFs to shape the T cell repertoire and, in particular, to regulate Th17 polarisation preferentially is noteworthy, as this subset plays essential roles in the immunopathology of inflammatory and autoimmune diseases^23^ and down-regulating of Th17 responses has therapeutic potential in these cases. Despite their potential to dampen pro-inflammatory Th17 responses, EFs also reduced the secretion of the anti-inflammatory IL-10. The significant decrease of IL-10 levels, but not FoxP3^+^ cells, in cultures following EF exposure indicates EF-specific effects on signalling events underpinning cytokine secretion, but not necessarily regulatory T cell polarisation and IL-10 production could be from alternative T cell types^24^.

Exactly how extracellular electrical cues are relayed into intracellular signals is largely unknown but influences on the distribution or conformation of membrane receptors have been suggested^25^. In a previous study EF-mediated cathodal migration of human lymphocytes was associated with activation of intracellular signalling by ERK kinases and Akt, which are also involved in chemoattractant receptor signalling^3,26^, but, unlike chemotaxis, no robust, immediate calcium flux was observed with EF exposure. We deliberately determined the effects of EF exposure on the later downstream events (18 h) induced by anti-CD3/CD28 antibody activation that result in T cell activation/polarisation, rather than the influence of immediate (1 h) EF induced signaling *per se* that were analysed previously^3^. At the timepoint examined, only pSTAT3 showed a consistent and significant downregulation following anti-CD3/CD28 antibody activation / EF exposure. The early EF induced changes in PI3K (Akt) and Erk signaling reported by Lin *et al*. were not evident at the later time point in our study or may have been masked by the additional anti CD3/CD28 induced T cell activation signalling. The selective interference we observed on the STAT3 signaling cascade could explain the decrease in production of IL-10, IL-17 and RORγt, which in signaling terms are located downstream of the IL-6 and IL-23 receptors. Th17 polarisation is normally driven by exposure to STAT3 activating cytokines e.g. IL-6, IL-23 and this pathway plays a critical role in Th17 cell development, which is impeded significantly in STAT-3 deficient T cells^27^. In our analysis of the effects of EF exposure on antigen-driven activation of T cell polarisation, LPS was used as a stimulus to induce production of Th polarising (STAT3-activating) cytokines, such as IL-6 and IL-23. The fact that EF exposure impairs STAT3 activity and dampens Th17 polarisation, which is principally driven by STAT3, provides a potential mechanism by which EFs modulate T cell polarisation. This inhibitory effect of EF on STAT3 activation is also relevant to the observation that EFs decrease the antigen-specific induced upregulation of CD25, the IL-2R alpha chain. Patients with loss-of-function mutations in STAT3 exhibit decreased expression of CD25^19^. This could link the observation that EF exposure impairs STAT3 activation to impaired T cell proliferation.

Naturally occurring EFs are present where epithelial barriers are disrupted and EFs accelerate healing *in vivo*. Clinical devices have now been used to apply synthetic EFs to accelerate healing processes through enhanced cell migration and improved angiogenesis. Given our results convincingly demonstrating that exposure to a physiological strength direct current EF cause a significant inhibitory effect on T cell proliferation and activation, one intriguing question is whether these synthetically applied EFs can therapeutically change the outcome of clinical disorders where T cell activity is dysregulated e.g. to dampen autoimmune lesions. Lin *et al*. have already demonstrated endogenous T cells actively migrate toward the cathode of a physiological strength applied EF in the skin of mouse ears confirming that they are responsive to EFs *in vivo*^3^. We now suggest, based on the differential effects of EF in suppressing Th17 differentiation, that future treatments based on EF application to limit Th17 inflammatory responses could be applied to specific tissues and organs that are sites of immune pathology, for example psoriasis.

In conclusion, our data contribute to the molecular mechanisms by which EFs direct T lymphocyte behaviour and provide a novel signalling paradigm in wound healing, tissue regeneration and immune-mediated diseases.

## Materials and Methods

### Cell isolation

Ethical approval for blood sampling from consenting healthy volunteers was granted by the Ethics Review Board of the College of Life Science & Medicine, University of Aberdeen and carried out in accordance with the relevant guidelines and regulations. Informed consent was obtained from all participants. Blood samples were collected into EDTA vacutainers (BIO-Greiner). Peripheral Blood Mononuclear Cells (PBMCs) were isolated by density gradient centrifugation using lymphoprep (Axis-Shield) according to manufacturers’ recommendation. T-cells were positively selected from PBMCs by magnetic-activated cell sorting (MACS), using anti CD2^+^ MicroBeads (Miltenyi Biotec)^17^. Cell purity was confirmed by flow cytometry and was routinely ≥ 98%.

### Electric field application

Custom-designed chambers for application of EF were prepared based on a modified version of that previously described^15,28,29^. In brief, glass coverslips were used to create a 50 × 10 × 0.2 mm channel within a 9 cm tissue culture dish. Discrete medium reservoirs were formed at both ends of the channel using silicone grease. Silver/silver chloride electrodes were inserted into Steinberg’s buffer reservoirs and connected to the chambers through 2% agar salt bridges. The EF was determined by measuring the voltage across the 50 mm channel with a voltmeter and adjusted using variable resistors. Isolated human T cells were seeded into the central channel prior to the application of EFs. T cells were exposed to EFs at 150 mV/mm for 4 hours. In selected experiments requiring high‐resolution imaging, ∼0.4 × 10^6^ T cells were seeded in µ‐Slide I ibiTreat flow chambers (80106; ibidi, Germany). These flow chambers are of a similar design as the custom EF chambers (channel dimensions, 50 × 5 × 0.4 mm), and the EF was applied as described above. EF exposure in both chamber types show equivalent effects on cell behaviours^15^.

### Cell viability

To determine their viability, T cells were stained with Annexin V dye conjugate to determine apoptotic cells and ToPro3 (Thermo Fisher Scientific) to determine dead cells. T cells were re-suspended in dyes as recommended by the manufacturer and incubated for 20 min on ice. Cells were washed and centrifuged at 350 x g for 8 min and analysed by flow cytometry on a LSRII (BD Biosciences) using Diva Software. Analysis of flow cytometry data was performed using FCS Express (De Novo).

### Activation of PBMC and exposure to EF

Isolated PBMC (2.5 × 10^6^) in 400 µL DMEM 5% serum were placed into the middle of the EF chamber and were incubated for 2 hours to enable attachment of adherent cells. Non-adherent cells were removed and stimulants (1 µg/mL LPS (OIII:B4 E.coli, Sigma Aldrich) and recall antigen, 5 µg/mL PPD; Statens Seruminstitute, Copenhagen) were added^17^. Non-adherent cells were transferred back into their respective assembled chambers in a total volume of 2.5 ml. EFs were applied at 150 mV/mm for 4 hours. Cells were washed out of the chamber on day 1 (36 hours) and cultured for 5 days (unless otherwise stated) and analysed as described.

### Activation of T cells and exposure to EFs

UV-irradiated EF chambers were coated with 5 µg/ mL anti-CD3 (eBioscience, clone: OKT3) in bicarbonate buffer for 1 hour. T cells (5 × 10^6^ cells in 5 mL complete DMEM) were stimulated with 2 µg/mL of α-CD28 (BD Biosciences, clone: CD28.2) then activated T cells were transferred into the EF chambers and EFs were applied immediately. Following EF-exposure (4 hours), the CD3/CD28-activated T cells were cultured within the chamber for 3 days (unless otherwise stated) and analysed as described.

### Cell proliferation

PBMC or T cells collected from EF chambers were exposed to 11.56 kBq/well [^3^H]-thymidine (Perkin Elmer) followed by incubation at 37 °C with 5% CO_2_ for 6 hours. DNA of cells was harvested onto glass fibre-filtermats (Perkin Elmer) by vacuum-manifold. Incorporation of [^3^H]-thymidine into the DNA of daughter cells was measured as radioactive decay in counts per minute (CPM) with a Trilux-Beta-counter LKB-WALLAC (Turku)^18^. Measured counts were corrected to background levels and presented as corrected counts per minute (CCPM). CCPM correlates to relative levels of proliferation of non-adherent cells (T cells) in cultures. In selected experiments division tracking in PPD/LPS activated PBMCs was performed with CellTrace Violet Cell proliferation kit^30^ (Thermofisher Scientific) as outlined by the manufacturer. PBMCs were co-stained with BB515 labelled anti-CD3 antibody (BD Bioscience) to identify T cells and dead cells were excluded by staining with fixable viability dye eFluor780 (eBioscience). To determine the expression of CD25, PBMCs were treated as above and additionally stained with BV711 labelled anti CD25 antibody (BD Biosciences). The labeled cells were acquired for flow cytometric analysis using a BD LSR Fortessa flow cytometry analyzer (BD Biosciences) and data were analyzed by FlowJo software (v10.4.2.Treestar Inc). The change in division index (average number of cell divisions that a cell in the original population has undergone) and the proliferation index (total number of divisions divided by the number of cells that went into division) was analysed under the Proliferation platform in FlowJo.

### T cell migration

Lymphocytes were plated on fibronectin and exposed to an EF (50 or 150 mV/mm) or with no EF at 37 °C in a temperature-controlled chamber on an inverted microscope stage. Serial time-lapse images were recorded every 30 seconds using a Zeiss Axiovert 200 M microscope and Zeiss Axiovision imaging software. Cellular migration was tracked using ImageJ software (*Manual Tracking* (NIH) and *Chemotaxis Tool* Plugins, Ibidi, Germany). Cell migration (100 cells per condition) was recorded by tracking nuclear displacement between frames over 15 minutes and only tracked cells that remained in the image frame over this time period and whose path could be determined unambiguously throughout recording were analysed^11^. The directedness of cell migration was defined as cosine θ, where θ is the angle defined by the EF vector (horizontal) and the line of Td (Td represents the straight-line distance between the position of the cell at the start (T = 0) and the end (T = 15 minutes) of the migration assay)^15^. The EF was set up with the anode on the right and cathode on the left of the electrotaxis chambers. The cosine θ values lie between −1 and +1 and are presented as the mean directedness index of all events. Therefore, a cell moving directly along the field lines toward the cathode would have a directedness of −1, whereas a cell moving directly to the anode would have a directedness of +1; a mean value close to 0 indicates random migration.

### Determination of T cell subsets

For the detection of cytokine-secreting T cells, their secretion was inhibited by addition of 2 mM monensin solution (eBiosciences) for 10 hours prior to T cell harvesting. T cells were fixed and permeabilized using the FoxP3-staining buffer kit (eBiosciences, 00–5523) according to manufacturers´ instructions. Extracellular markers were stained then T cells (including unstained controls) were re-suspended in 100 µL of Fixation/Permeabilization reagent and were incubated for 20 min on ice. The master mix for intracellular staining was prepared with 2% mouse serum in permeabilization buffer and relevant antibodies (Supplementary Table 1). The cell pellets were re-suspended in 100 µL of Master Mix. Unstained controls were re-suspended in permeabilization buffer. T cells were incubated for 30 min on ice in the dark. Fluorescently labelled cells were acquired on a LSRII (BD Biosciences) using Diva Software. Compensation was set-up using single positive stained beads or cells and voltages were adjusted accordingly. Compensation was automatically calculated by the Diva Software. The events counted were dependent on the number of cells available and were generally between 20000 and 100000 acquired events per sample and identical numbers were acquired within one experiment. Analysis of flow cytometry data was performed using FCS Express (De Novo).

### Cell culture supernatant measurement of cytokines

Supernatants were collected from PBMCs or T cells after 5 days of culture. Concentrations of IFNγ and IL-10 (matched Ab-pairs BD Biosciences) and IL-17A (matched Ab-pair eBioscience) were determined by ELISA using conditions recommended by the manufacturer. 96-well/plates (NUNC) were coated with primary antibody at 2 µg/mL in bicarbonate buffer for 1.5 hours at 37 °C followed by blocking of non-specific binding sites with 2% bovine serum albumin (BSA) in PBS for 1 hour at 37 °C. Plates were washed twice and 100 µL/well of culture supernatant was loaded onto ELISA plates in duplicates and incubated for 1.5 hours at room temperature. Secondary antibody was applied at 1 µg/mL (IFNγ and IL-10) and 0.5 µg/mL (IL-17A) in 0.2% BSA PBS for 1.5 hour at room temperature. Plates were incubated with 100 µL/well ExtrAvidin (1:10000) (Sigma Aldrich) for 1 hour followed incubation with phosphatase substrate 1 mg/mL (Sigma Aldrich) until optimal development was achieved. Due to the unstable nature of IL-4, a cell ELISA determined IL-4 concentrations. Standards or cell suspensions were loaded onto IL-4 precoated plates and incubated overnight at 37 °C, 5% CO_2_. Substrate development was measured with Labsystem Multiscan MS plate reader and cytokine levels were calculated from standard curves generated with Genesis software^13^.

### Western blotting

T cells (9 × 10^5^) were seeded in custom EF chambers and then exposed to 150 mV/mm for 4 h then left for 18 h to induce activation. Protein lysates were prepared from T cells using lysis buffer containing a phosphatase and protease inhibitor cocktail. A total of 20 µg protein was separated by SDS-PAGE and transferred to Hybond^TM^-P PVDF membrane (Amersham, GE Healthcare) for Western blot analysis with specific primary antibodies for Akt and p-Akt ser473; STAT1 and p-STAT1 (tyr 701), STAT3 and p-STAT3 tyr705; p38 and p-p38 thr180/tyr182; ERK and p-ERK thr202/tyr204 (Cell Signaling Technology) and β-actin (Sigma-Aldrich). Immunolabelled proteins were detected using appropriate horseradish peroxidase-conjugated secondary antibodies, followed by visualization with ECL Prime Western Blotting Detection Reagent (GE Healthcare). Bands were normalized to β-actin. The phosphoprotein and total protein as well as β-actin loading control was detected on the same blot. Following analysis of phosphoprotein, the blot was stripped and reprobed for total protein and β-actin loading control in the same run.

### Statistical analysis

Statistical analysis was performed with Prism 5.0 (GraphPad). To normalise levels of T cell proliferation and cytokines the background levels of cultures without stimulation and antigen were subtracted from corresponding data. Logarithm of normalised data was taken and data were tested for normal distribution. In the event of normal distribution a Students paired t-test was performed. In the absence of normal distribution significance was based on Wilcoxon-Signed-Rank-Test.

## Supplementary information

Supplemental video 1

Supplementary information

## Data Availability

All data generated or analysed during this study are included in this published article (and its Supplementary Information files).
