# Supplementary material for: Physiological strength electric fields modulate human T cell activation and polarisation
Source: Sci Rep. 2019 Nov 26;9:17604. doi: 10.1038/s41598-019-53898-5 (PMC6879562; doi:10.1038/s41598-019-53898-5)
Supplement: Supplementary file 2 — Supplementary information [file 41598_2019_53898_MOESM2_ESM.pdf]

Supplementary information

Physiological strength electric fields modulate human T cell activation and polarisation

Christina E Arnold, Ann Rajnicek, Joseph I Hoare, Swechha Mainali Pokharel, Colin D McCaig, Robert N Barker, Heather M Wilson*

School of Medicine, Medical Sciences & Dentistry, University of Aberdeen, Foresterhill, Aberdeen, AB25 2ZD, UK

***Corresponding author:** Dr Heather M Wilson, School of Medicine, Medical Sciences & Nutrition, University of Aberdeen, Foresterhill, Aberdeen, AB25 2ZD, Scotland, UK

Tel: +44 1224 437965, **E-mail:** h.m.wilson@abdn.ac.uk

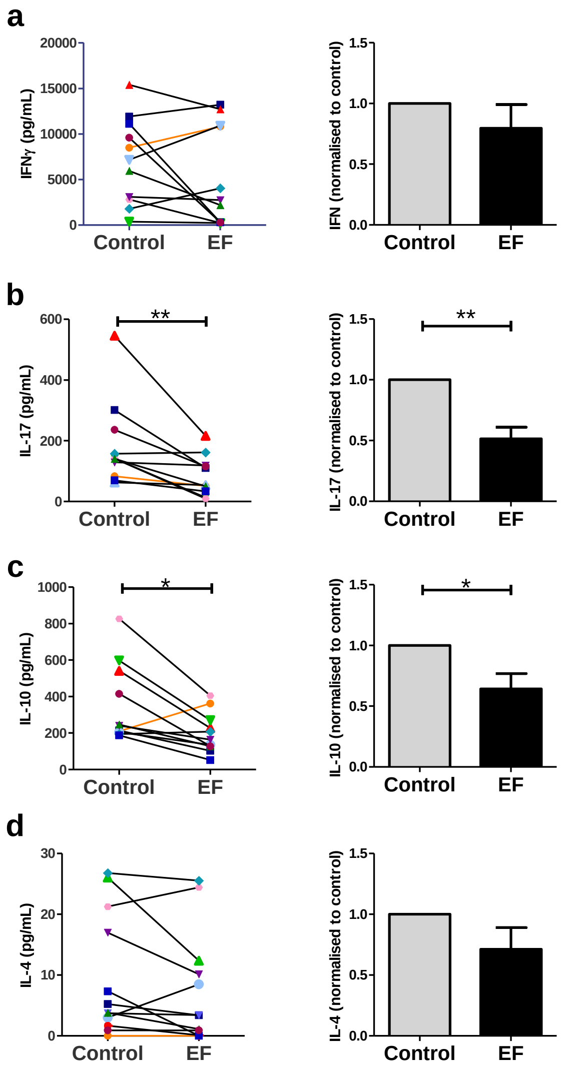

Supplementary Figure 1: EFs attenuate PBMC-derived cytokine secretion. Human PBMCs (2.5x10^{6}) were activated by PPD recall antigen (5 µg/ml) and LPS (1 µg/ml), with or without exposure to EF 150mV/mm for 4h. (a) Supernatants were analysed for cytokine production by ELISA, 5 days post EF application. Data plotted represent individual donor values and right panels represent the normalised data determining the ratio of EF-exposed cells to control cells for each donor; mean values ± SEM. *p < 0.05.

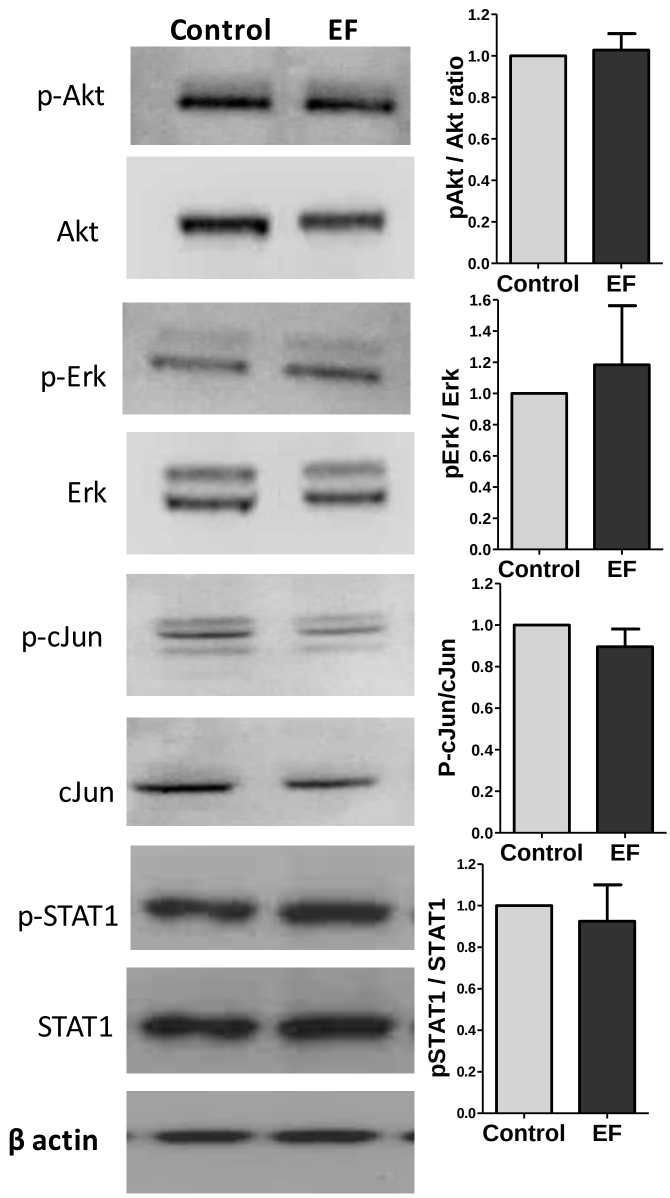

Supplementary Figure 2: EF exposure does not cause a consistent change in Akt, Erk, cJun or STAT1 activation. T cell derived protein was isolated 18 hours following 4h EF-exposure (150mV/mm) or non-EF exposed cells and analysed for Akt, Erk, cJun or STAT1 activity and ß-actin expression by Western Blotting. The ratio of phosphorylated (activated) to total transcription factor band intensity, was determined by densitometry. Shown as mean ± SEM ratios of independent experiments from 4 individual human T cell preparations. Blots for the phosphoprotein and total protein shown, are from the same gel. The blots were stripped after probing for phosphoprotein and reprobed with total protein. pAKT/AKT and pERK/ERK and β actin were analysed on the same gel due to distinct differences in molecular weight. P-cJun/cJun and p-STAT1/STAT1 were analysed on separate gels.

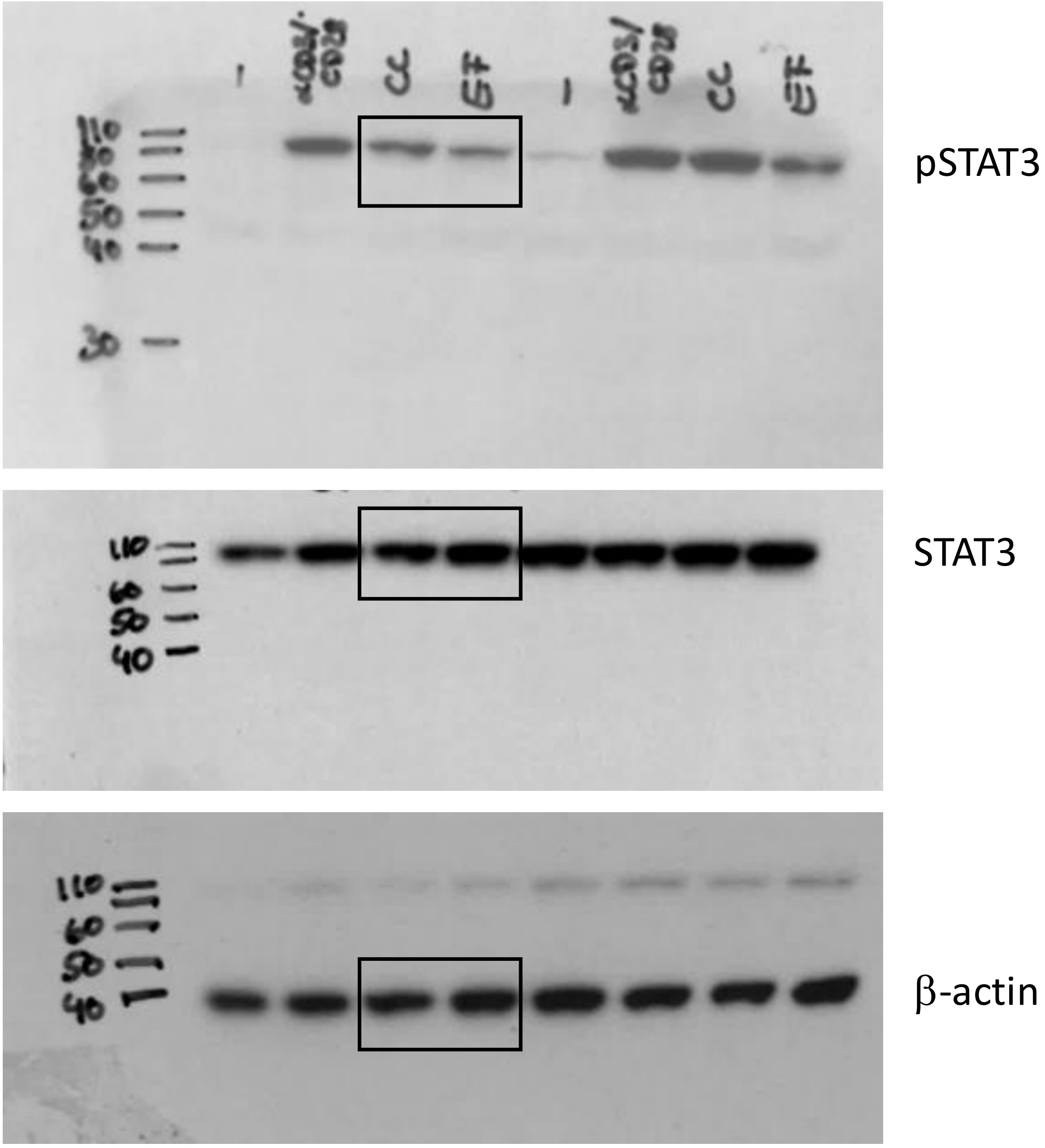

Supplementary Figure 3: EF exposure results in a decrease in STAT3 phosphorylation in T cells. T cell derived protein was isolated 18 hours following 4h EF-exposure (150mV/mm) and analysed for STAT3, pSTAT3 and β-actin expression levels by Western Blotting. Blots shown are from the same gel. The blots were stripped after probing for pSTAT3 and reprobed for total STAT3 and β-actin. The lower gel shows total STAT3 and β-actin on the same blot. Full scan images of immunoblots for main Figure 6. Lane 1: non-activated cells; lane 2: activated control (T cell preparation 1); lane 3: EF control (T cell preparation 1); lane 4 EF exposed cells (T cell preparation 1); lane 5 non-activated cells; lane 6 activated control (T cell preparation 2); lane 7: EF control (volunteer T cell preparation 2); lane 8: EF exposed cells (T cell preparation 2).

Epitope	Conjugate	Manufacturer	Code	Clone	Isotype
IL-17	Alexa Fluor®647	eBiosciences	51-7177	eBio17B7	IgG2a, κ(rat)
RORγt	PE	eBiosciences	12-6988	AFKJS-9	IgG2a (rat)
T-bet	PerCPCy®5.5	eBiosciences	45-5825	4B-10	IgG1, κ
GATA-3	eFluor®660	eBiosciences	50-9966	TWAJ	IgG2b (rat)
Fox-P3	AlexaFluor®488	eBiosciences	53-4777	236A/E7	IgG1, κ
CD3	BB515	BD Biosciences	564465	UCHT-1	IgG1, κ
CD25	BV711	BD Biosciences	563159	2A3	IgG1, κ
Isotype	PE	eBiosciences	12-4321		IgG2a (rat)
Isotype	PerCPCy®5.5	eBiosciences	45-4714		IgG1, κ
Isotype	eFluor®660	eBiosciences	50-4031		IgG2b (rat)
Isotype	FITC	BD Biosciences	551954		IgG1, κ

Supplementary Table 1 Fluorescently labelled antibodies used for intracellular staining; Host species of antibodies is mouse unless stated otherwise
